# Comment on “utilizing Raman spectroscopy for urinalysis to diagnose acute kidney injury stages in cardiac surgery patients”

**DOI:** 10.1080/0886022X.2024.2437134

**Published:** 2024-12-03

**Authors:** Ivan A. Bratchenko, Lyudmila A. Bratchenko

**Affiliations:** Laser and Biotechnical Systems Department, Samara National Research University, Samara, Russia

Dear Editor,

Raman spectroscopy offers a way for accurate, conventional and label-free analysis of tissues and biofluids in real time mode [1–5]. The precise interpretation of spectral data and accurate identification of different diseases are made possible by the combination of Raman spectroscopy and machine learning algorithms [1]. Simultaneously, the complex nature of spectral data and current machine learning algorithms frequently result in the development of inaccurate and overestimated classification models [4]. As example, there are studies that even offer noninvasive estimation of blood glucose level in human, but such approaches appears overoptimistic due to incorrect machine learning approaches [5].

The paper by Sharma et al. [6] demonstrates combination of conventional Raman spectroscopy and PLS-SVM (partial least squares - support vector machine) for the classification of urine of acute kidney injury (AKI), and non-AKI group. The authors achieved 93.3% accuracy, however, the presented results may be treated incorrectly due to the drawbacks of the proposed validation procedure for classification models.

Sharma et al. proposed to utilize k-fold cross-validation and divided analyzed data into training and test. Moreover, the authors repeated this process numeral times and provided averaged values of performances. Generally, utilization of cross-validation data split into training and test is an adequate strategy that prevents classification models from overfitting. However, in the proposed pipeline of spectral data analysis, the authors, prior to data splitting, perform a PLS analysis of the whole spectral dataset and define a shape of loading vectors (LVs) that will further be utilized in the SVM classifier. In such approach information about all spectra in the analyzed dataset is already presented in the obtained LVs (as these LVs were constructed with the utilization of the whole dataset). Thus, despite further separation of data into training and test the model already knows what data to expect in the test set if it operates with LVs obtained from the whole dataset. In this regard, it may be advised to Sharma et al. to construct LVs only on the data from the training set and next evaluate models performance on the truly unseen part of the data in the test set.

In addition, during PLS or PCA (principal component analysis) utilization, it is very important to evaluate not only the accuracy of the obtained classifier vs. the number of utilized LVs, but evaluate an obtained LVs shape [1]. High-order LVs will contain mostly noise components, while first LVs will demonstrate distinct Raman bands. Approaches like variable importance in projection estimation and comparison of training and validation data [1] may be helpful in the task of LVs number limitation. In this regard, it is advised to demonstrate and evaluate LVs shape in order to exclude noise information from the analysis. Moreover, Sharma et al. presented only mean values of the obtained performances and only data for model tests. In multiple data splits, it is important to evaluate not only a mean value, but also a standard deviation, that may highlight the actual limits of the obtained performance. In data division, it is important to demonstrate full results for training, validation, and test. In this case, the readers may evaluate the performances of the proposed models during each step of model construction.

In general, the commented paper provides novel insight into the possibilities of Raman spectroscopy combined with PLS-SVM to differentiate AKI and non-AKI samples with liquid biopsy of human urine. However, in order to confirm that the obtained high performance values are correct, the authors of the commented paper should demonstrate data for LVs constructed solely on the training data portion.
